# Photoperiod Conditions Modulate Serum Oxylipins Levels in Healthy and Obese Rats: Impact of Proanthocyanidins and Gut Microbiota

**DOI:** 10.3390/nu15030707

**Published:** 2023-01-30

**Authors:** Verónica Arreaza-Gil, Javier Ávila-Román, Iván Escobar-Martínez, Begoña Muguerza, Manuel Suárez, Anna Arola-Arnal, Cristina Torres-Fuentes

**Affiliations:** 1 Nutrigenomics Research Group, Department of Biochemistry and Biotechnology, Universitat Rovira i Virgili, 43007 Tarragona, Spain; 2 Molecular and Apply Pharmacology Group (FARMOLAP), Department of Pharmacology, Faculty of Pharmacy, University of Sevilla, 41012 Sevilla, Spain

**Keywords:** biological rhythms, cafeteria diet, GSPE, obesity, resolvins

## Abstract

Seasonal rhythms are emerging as a key factor influencing gut microbiota and bioactive compounds functionality as well as several physiological processes such as inflammation. In this regard, their impact on the modulation of oxylipins (OXLs), which are important lipid mediators of inflammatory processes, has not been investigated yet. Hence, we aimed to investigate the effects of photoperiods on OXLs metabolites in healthy and obesogenic conditions. Moreover, we evaluated if the impact of proanthocyanidins and gut microbiota on OXLs metabolism is influenced by photoperiod in obesity. To this purpose, Fischer 344 rats were housed under different photoperiod conditions (L6: 6 h light, L12: 12 h light or L18:18 h light) and fed either a standard chow diet (STD) or a cafeteria diet (CAF) for 9 weeks. During the last 4 weeks, obese rats were daily administered with an antibiotic cocktail (ABX), an oral dose of a grape seed proanthocyanidin extract (GSPE), or with their combination. CAF feeding and ABX treatment affected OXLs in a photoperiod dependent-manner. GSPE significantly altered prostaglandin E2 (PGE2) levels, only under L6 and mitigated ABX-mediated effects only under L18. In conclusion, photoperiods affect OXLs levels influenced by gut microbiota. This is the first time that the effects of photoperiod on OXLs metabolites have been demonstrated.

## 1. Introduction

Biological rhythms have been shown to affect several metabolic and physiologic processes [1,2]. Thus, many of these processes follow circadian oscillations whose disruption has been associated with an increased risk of obesity and other related pathologies [3]. In fact, it has been demonstrated that seasonal rhythms can influence the risk of obesity development [4]. In this context, studies carried out in animals exposed to different photoperiods, which mimic the differences in day length associated with seasonal rhythms, displayed higher body weight (BW) gain and white adipose tissue accumulation under longer photoperiod conditions (summer) [4,5]. Among humans, the link between seasonal rhythms and obesity has only been described in observational studies. Thus, it has been observed that children gained more weight in summer and early fall compared to winter [6,7]. This fact has been strongly associated with the lifestyle, as changes in the sleep/wake cycles or feeding behaviors promote circadian misalignment, which could lead to altered patterns of seasonal rhythms [8].

Interestingly, seasonal rhythms have also been shown to influence immune system responses in most vertebrate mammals [9]. In this context, photoperiods regulate immune activities through melatonin signaling and neuroendocrine pathways regulation, adjusting energetic investment according to environmental conditions and thus ensuring survival [10,11]. Hence, it has been hypothesized that mammals increase their investment in immune function in response to the short photoperiod (winter), as it increases the risk of infection and diseases due to its stressful conditions of temperature and food availability compared to other seasons of the year [10]. Numerous studies in rodents have demonstrated this photoperiodic immune response [9]. For instance, Siberian hamsters exposed to short photoperiod showed higher numbers of total leukocytes, lymphocytes, T cells, and natural killer (NK) cells than those subjected to long photoperiod [12]. Similarly, Wistar rats (non-photoperiodic animals) had a higher numbers of leukocytes and an increased proinflammatory cytokine response under short photoperiods [13]. Nonetheless, the impact of photoperiod on human immune system response is much less well understood and further investigations are needed. Even so, some evidence supports that humans also follow photoperiodic immune patters. Thus, healthy individuals showed seasonally different responses in circulatory cytokines levels after endotoxin (lipopolysaccharides, LPS) administration, anti-inflammatory cytokine production being lower in summer compared to winter [14]. Moreover, a study in healthy humans showed that NK cells, interleukin 6, and peripheral blood monocular cells proliferation were increased under short days [15].

Oxylipins (OXLs) are potent bioactive lipid metabolites involved in the activation and resolution of the inflammatory response [16]. These metabolites are produced as a result of the oxygenation of dietary polyunsaturated fatty acids (PUFAs) by cyclooxygenase (COX), lipoxygenase (LOX), and cythochrome P450 (CYP) enzymes [17]. Most OXLs are generated in the initial phase of inflammation from arachidonic acid (ARA, 20:4*n*-6) [18]. ARA-derived OXLs can be both proinflammatory, such as prostaglandins, leukotrienes, and thromboxanes, or anti-inflammatory, such as lipoxins [19]. Moreover, OXLs can also be synthetized from both dietary eicosapentanoic acid (EPA, 20:5*n*-3) and docosahexaenoic acid (DHA, 22:6*n*-3), generating mostly anti-inflammatory OXLs such as resolvins, protectins, and maresins [20,21]. In addition, OXLs can be produced by other fatty acids such as from linoleic acid (LA), constituting more than one-half of OXLs present in tissues [18]. Thus, LA can be converted to di-homo-gamma-linoleic acid (DGLA, 20:3, *n*-6), which can be transformed to ARA. Hence, OXLs play a crucial role in the maintenance of inflammatory homeostasis, being of significant relevance in several disorders associated to chronic inflammation such as obesity [22]. In fact, altered OXLs profile has been linked to obesity in both animals and human subjects, suggesting that these metabolites can be used as obesity biomarkers and as a target for the prevention of this disorder [23,24,25]. This is important as obesity and obesity-related disorders prevalence continues increasing worldwide affecting more than two billion people, and being a major predisposing factor to several comorbidities such as type 2 diabetes, hypertension, nonalcoholic fatty liver disease, cardiovascular diseases, and several types of cancer [26,27,28]. Therefore, OXLs are emerging as potential biomarkers for these diseases and understanding the factors affecting their metabolism is of increasing interest in the last years. However, the influence of photoperiod on these lipid mediators has not been investigated yet.

Regarding factors affecting OXLs, gut microbiota has been demonstrated to influence these metabolites via metabolism of PUFAs which, as mentioned above, are OXLs precursors [29]. In fact, a strong correlation between these gut microbes and plasma OXLs profile has been recently reported by our group and others [25,29]. Moreover, bioactive compounds seem also to impact the OXLs profiles. Thus, polyphenols have been shown to affect OXLs biosynthesis in human neutrophils [30]. In addition, it was observed that dietary supplementation with polyphenol-rich juice led to changes in urine OXLs profile in elite triathletes [31], and that blueberry consumption mitigated OXLs generation during the recovery phase after physical exercise in humans [32]. Therefore, the gut microbiota composition and the consumption of polyphenols could be a therapeutic target to modulate the levels of OXLs in obesity.

Interestingly, our group reported that biological rhythms can influence both gut microbiota [33] and polyphenols bioactivities [34,35]. Remarkably, we recently reported that photoperiod can influence the functionality of one grape seed proanthocyanidin extract (GSPE) via gut microbiota [36]. This fact is relevant since GSPE has shown anti-obesity properties [37,38,39]. However, the interaction among photoperiod, gut microbiota, and polyphenols on OXLs levels, and its role in obesity has not been elucidated yet.

Taking all together, this study aimed to investigate the effect of different photoperiods on OXLs serum profile in healthy and obese rats, as well as the impact of gut microbiota and GSPE. Moreover, to further investigation, we analyzed the effects on LA, which can also act as OXLs precursor as well as on oleic acid (OA), a non-essential monosaturated fatty acid that has been shown to impact PUFA content in peripheral tissues such as liver and adipose tissue, influencing the levels of bioactive OXLs [40]. In particular, we evaluated their effects on prostaglandins 6-keto Prostaglandin F1α (6-keto PGF1α) and Prostaglandin E2 (PGE2), resolvin D2 (RvD2), 15-epi Lipoxin A4 (15-epi LXA4), and the leukotriene B4 (LTB4). 6-keto PGF1α is a marker of prostacyclin (PGI2) biosynthesis, which has been described as anti-inflammatory or immunosuppressive [41], while PGE2 has been described as proinflammatory [42]. RvD2 is involved in the resolution of inflammatory processes and has been demonstrated to prevent different inflammatory conditions, including allergic reactions, chronic low-grade inflammation of adipose tissue and atherosclerosis [43]. 15-epi LXA4 has also been reported to exert anti-inflammatory effects in different tissues [44,45]. LTB4 is a potent proinflammatory lipid mediator that has also been shown to promote insulin resistance in obese mice [46].

## 2. Materials and Methods

### 2.1. Experimental Design

**Figure 1:**
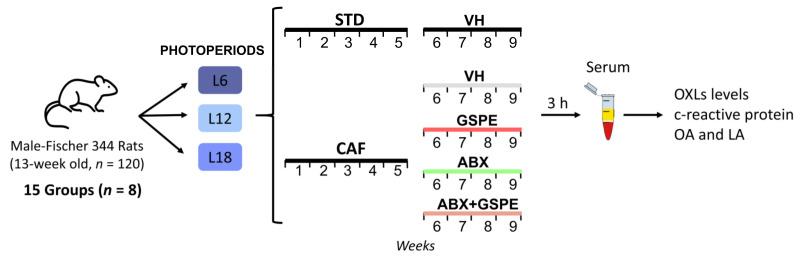
Experimental design. A total of 120 13-week-old male Fischer 344 rats were pair-housed under three different photoperiods (6, 12, or 18 h of light per day) and fed a STD or CAF for 9 weeks. During the last 4 weeks, animals were daily administered with either an oral dose of GSPE (grape seed proanthocyanidin extract) (25 mg/kg) dissolved in a solution of water and condensed milk (5:1, VH), or a combination of GSPE and an antibiotic cocktail (ABX) in drinking water (ampicillin: 0.5 g/L, vancomycin: 0.25 g/L, imipenem: 0.125 g/L). Vehicle and ABX-treated animals were included as controls. L6: 6 h light/18 h darkness; L12: 12 h light/12 h darkness; L18: 18 h light/6 h darkness; STD: standard chow diet; CAF: cafeteria diet; VH: vehicle; OXLs: oxylipins; OA: oleic acid; LA: linoleic acid.

The experimental design used has been previously described by our group [33,36]. Thus, 120 13 weeks-old male Fischer 344 rats (Javier Laboratories, Le Genest-Saint-Isle, France) were pair-housed in standard conditions (22 °C, 65% relative humidity and 12:12 h light/dark cycle) with ad libitum access to water and standard chow diet (STD) for one week. After this acclimation period, each rat was weighted and randomized distributed into three different light-dark cycles (photoperiods) for 9 weeks to mimic seasonal day lengths (*n* = 40 per photoperiod): short photoperiod (L6, 6 h light/18 h darkness), standard photoperiod (L12, 12 h light/12 h darkness), or long photoperiod (L18, 18 h light/6 h darkness). In each photoperiod, rats were further randomly assigned to five groups depending on the diet administered during the 9 weeks of the experiment and the treatment received for the last four weeks (*n* = 8 per group) (Figure 1):

1. STD-fed rats (72% carbohydrate, 8% lipid, and 19% protein; Safe-A04c, Rosenberg, Germany) receiving a daily oral dose of vehicle (VH, condensed milk diluted with water in 1:5 proportion).
2. Cafeteria diet (CAF)-fed rats receiving a daily oral dose of VH.
3. CAF-fed rats administered a daily oral dose of GSPE (25 mg/kg BW dissolved in VH).
4. CAF-fed rats receiving an antibiotic cocktail (ABX) in drinking water.
5. CAF-fed rats receiving both a daily oral dose of GSPE and ABX in drinking water.

CAF rats had ad libitum access to the CAF diet that was freshly prepared every day. CAF diet was composed of highly palatable and energy-dense human foods. CAF included the following foods (grams per rat and per day): biscuits with pâté and cheese (15–17 g), bacon (7–10 g), ensaimada (pastry) (10–15 g), carrot (11–12 g), standard chow (20–25 g), and milk containing 22% sucrose (*w*/*v*) (58% CH, 31% lipid, and 11% protein). The ABX (0.5 g/L ampicillin, 0.250 g/L vancomycin, and 0.125 g/L imipenem; Discovery fine chemicals, UK) was freshly prepared every day and the animals were given free access to it in the drinking water. VH and GSPE dose was administered one hour after the light was turned on by allowing rats to drink it from the tip of a syringe. The 25 mg/kg GSPE dose can be translated from animals to human, corresponding to an intake of approximately 370 mg of GSPE per day for a 70 kg human [47,48].

BW was recorded weekly (Figure S1a). Intake was also recorded weekly and it is shown as total accumulated intake in Figure S1b. Animals were sacrificed by decapitation following last treatment dose and 3 h of fasting. The blood was collected from the neck, in non-heparinized tubes, incubated for 1 h at room temperature and immediately centrifuged at 1200× *g* for 15 min to collect the serum. The Animal Ethics Committee of the University Rovira i Virgili (Tarragona, Spain) and the Generalitat de Catalunya approved all the procedures (number reference 9495) and in accordance with the EU Directive 2010/63/EU for animal experiments.

### 2.2. GSPE

GSPE was provided by Les Dérives Résiniques et Terpéniques (Dax, France). According to the manufacturer, this phenolic-rich extract is mainly composed by monomers of flavan-3-ols (21.3%), dimers (17.4%), trimers (16.3%), tetramers (13.3%) and oligomers (5–13 units; 31.7%) of proanthocyanidins [36,49].

### 2.3. C-Reactive Protein Analysis

C-reactive protein levels were analyzed in serum samples using a rat C-reactive ELISA kit (Elabscience, Houston, TX, USA), according to the manufacturers’ instructions.

### 2.4. OXLs Serum Analysis

6-keto PGF1α, 15-epi LXA4, LTB4, PGE2, and RvD2 were analyzed in serum samples using Cayman Chemical’s AchE kits (Ann Arbor, MI, USA) according to the manufacturers’ instructions.

### 2.5. Fatty Acids Serum Analysis

LA (18:2*n*-6) and OA (18:1*n*-9) were analyzed from 100 µL of serum samples. Serum sample were deproteinized by the addition of 400 µL of methanol:water (8:2) containing internal standard mixture. The samples were vortexed and incubated at 4 °C for 10 min. Then, they were centrifuged at 4 °C for 10 min at 15,000× *g* rpm and supernatant was evaporated to dryness before compound derivatization (methoximation and silylation). The derivatized compounds were analyzed by GC-qTOF (model 7200 of Agilent, Santa Clara, CA, USA). The chromatographic separation was based on Fiehn Method, using a J&W Scientific HP5-MS (30 m × 0.25 mm i.d., 0.25 μm film capillary column) and helium as carrier gas using an oven program from 60 to 325 °C. Ionization was done by electronic impact, with electron energy of 70 eV and operating in full Scan mode.

Identification of LA and OA were performed using commercial standards and by matching their Electronic Impact mass spectrum and retention time to metabolomic Fiehn library (from Agilent, Santa Clara, CA, USA). After putative identification of these fatty acids, LA and OA were semi-quantified in terms of internal standard response ratio.

### 2.6. Statistical Analysis

Data are shown as mean ± standard error of mean (SEM) and were plotted using Graphpad Prism 8.0 software (Graphapad software Inc, San Diego, CA, USA). Statistical analyses were carried out using SPSS software (IBS SPSS statistics 25, Chicago, IL, USA). Normality and homogeneity of variance were evaluated by Shapiro–Wilk and Levene’s test respectively. BW gain, C-reactive protein, LA, OA, and OXLs were analyzed using two-way analysis of variance (ANOVA) to evaluate diet and photoperiod effect (Photoperiod x Diet) followed by LSD post hoc test and using three-way ANOVA to evaluate ABX, GSPE, and Photoperiod effect followed by LSD post hoc test. Statistical significances are subsequently depicted as follows: * indicating diet effect *p* < 0.05, # indicating ABX effect *p* < 0.05, $ indicating GSPE effect *p* < 0.05 and *ab* indicating photoperiod effect *p* < 0.05.

Principal component analysis (PCA) involving serum OXLs levels data were analyzed and plotted using MetaboAnalyst v.5.0 [50]. Missing values were replaced by the half of the minimum positive values in the data, assuming that most missing values are caused by low abundance metabolites.

Pearson’s rank-order correlation analysis between OXLs levels and LA and OA were carry out using Python script as previously described [25]. The script was developed using PyCharm software (v.2018.2.4, JetBrains s.r.o., Prague, Czech Republic) and Python version 3.7.7.

## 3. Results

### 3.1. OXLs Levels Are Affected by Photoperiods Conditions Depending on the Healthy or Obese Condition. CAF Effects Are Different Depending on the Photopeiod Condition

CAF-induced obese rats showed a significant higher BW gain, a higher intake, and increased levels of inflammation as measured by C-reactive protein serum levels compared to healthy rats (Figure S1). This fact indicates successful development of obesity and related inflammatory status with this experimental model using CAF feeding, independently of the photoperiod condition. Moreover, photoperiod effects were observed for BW gain in CAF-fed rats, being highest under L18 conditions, while no photoperiod effects were observed in STD-fed rats (Figure S1).

**Figure 2:**
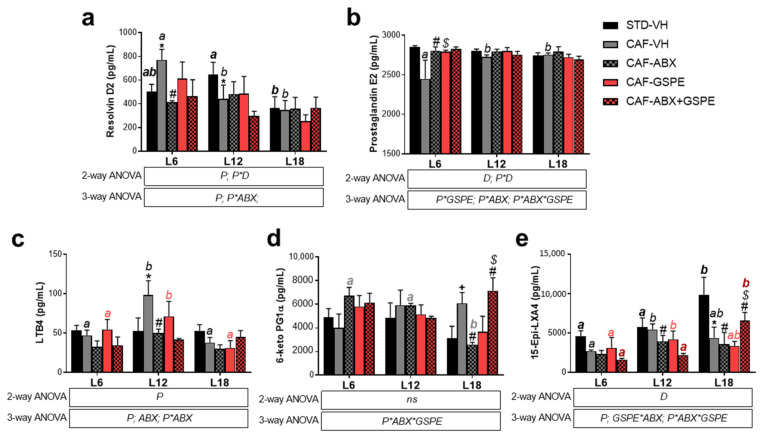
Diet, GSPE, and Photoperiod effect on OXLs levels. (**a**) 6-keto Prostaglandin F1α, (**b**) LTB4, (**c**) Prostaglandin E2, and (**d**) 15-epi LXA4levels; (**e**) RvD2 levels. * indicates significant diet effect between STD-VH and CAF-VH rats by 2-way ANOVA (factors: diet (D) and photoperiod (P)) followed by LSD post hoc test (*p* < 0.01); #, $, and *ab* letters indicate significant ABX, GSPE, and Photoperiod effect, respectively, analyzed by 3-way ANOVA followed by LSD post hoc test (*p* < 0.05); *ab* letters are shown in different color for each group. + indicates significant differences between STD- and CAF-fed rats analyzed by Student’s *t*-test (*p* < 0.05); Data are plotted as the mean ± SEM (*n* = 5).

**Figure 3:**
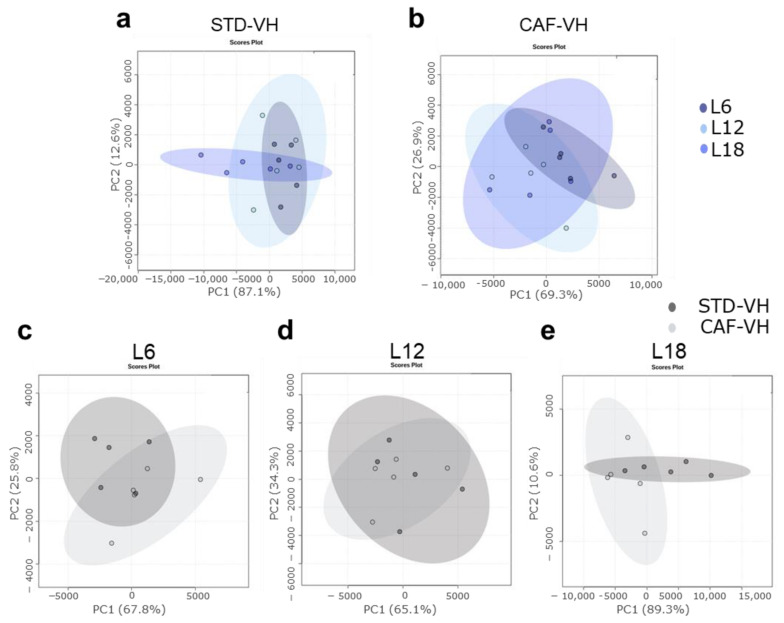
OXLs profile was influenced by the different photoperiod in healthy and obese rats and CAF feeding effect on OXLs profile was different according to the photoperiod exposure. (a to b): Photoperiod effect on (**a**): healthy rats (STD-VH); and (**b**): CAF-induced obese rats (CAF-VH) plotted as principal component analysis (PCA). (c to d): Diet effect in (**c**): L6; (**d**): L12; and (**e**): L18 conditions throughout PCA. (*n* = 5).

Photoperiod influenced the OXLs levels in both healthy and obese rats. Thus, healthy rats showed a photoperiod effect on RvD2, which showed higher levels under L12 compared to both L6 and L18, and on 15-Epi-LXA4, which showed higher levels under L18 compared to both L6 and L12 (Figure 2). Interestingly, a higher number of OXLs were affected by photoperiod in obese rats. In this context, obese rats showed higher levels of RvD2 under L6 compared to both L12 and L18, lower PGE2 levels under L6 compared to both L12 and L18, higher LTB4 levels under L12 compared to both L6 and L18, and lower levels of 15-epi-LXA4 under L6 compared to L12 (Figure 2). Furthermore, the influence of photoperiod on OXLs levels in healthy and obese rats were also analyzed using PCA. Thus, OXLs serum levels clustered differently in healthy rats housed under L18 conditions compared to those healthy rats housed under both L6 and L12 conditions (Figure 3a). Remarkably, this photoperiod effect was different in obese rats, which showed a higher separation under L6 (Figure 3b), suggesting that OXLs levels from healthy and obese rats are differently affected by photoperiod exposure.

Interestingly, the CAF effects on OXLs levels were different depending on the photoperiod conditions (Figure 2). Thus, RvD2 was significant increased in CAF-fed rats under L6 while it was decreased under L12, and no changes were observed under L18 compared to STD-fed rats (Figure 2a). Although it was not significant, PGE2 was decreased in CAF-fed rats only when housed under L6 conditions (Figure 2b). Furthermore, LTB4 was increased in CAF-fed rats compared to STD-fed rats only under L12 conditions while no changes were observed under L6 or L18 (Figure 2c). Moreover, 6-keto-PG1Fα was increased in CAF-fed rats under L18 while no changes were observed in L6 or L12 conditions (Figure 2d). Finally, 15-Epi-LXA4 was decreased in CAF-fed rats under L18 conditions while no changes were observed in L6 or L12 (Figure 2e). Additionally, the impact of CAF feeding on OXLs levels of rats housed under the different photoperiod conditions was also analyzed by PCA. PCA showed a higher effect of CAF feeding under long photoperiod conditions (PC1 = 89.3%) compared to both short (PCA1 = 67.8%) or standard (PCA1 = 65.1%) photoperiod conditions (Figure 3c,d), suggesting that the diet effect on OXLs levels is influenced also by the different photoperiod conditions.

### 3.2. Photoperiod Effects on OXLs Serum Levels in Obese Rats Is Influenced by Gut Microbiota

ABX treatment did not affect BW gain, intake, or C-reactive protein serum levels (Figure S1). Interestingly, ABX treatment abolished the photoperiod effect observed in OXLs levels in obese rats, commented above, suggesting that the photoperiod effect on OXLs levels is influenced by the gut microbiota (Figure 2). In addition, it is worth highlighting that ABX treatment altered OXLs serum levels differently depending on the photoperiod conditions. Thus, ABX significantly decreased RvD2 and increased PGE2 in rats housed under L6 conditions. Furthermore, in rats housed under L12 conditions, ABX significantly decreased LTB4 and 15-Epi-LXA4 levels. Finally, ABX-treated rats housed under L18 conditions showed a significant decrease in 6-keto PG1Fα and in 15-Epi-LXA4 levels (Figure 2). Thus, except by the increase of PGE2 levels by ABX, an overall decreased level of OXLs was found by ABX depending on photoperiod conditions, these levels being similar to those observed in healthy rats, except in 15-Epi-LXA4 under L18 conditions (Figure 2a–e).

**Figure 4:**
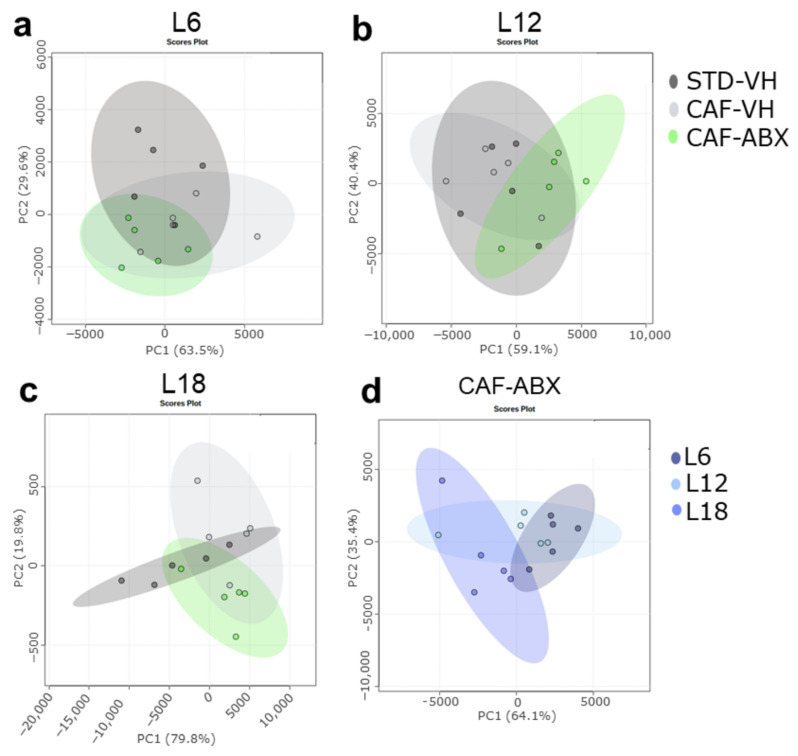
OXLs profile was influenced by the gut microbiota in a photoperiod-dependent manner. (**a**–**c**): ABX effect in (**a**): L6; (**b**): L12; and (**c**): L18 conditions throughout Principal Component Analysis (PCA); (**d**): Photoperiod effect on CAF-ABX-treated rats. (*n* = 5).

In addition, PCA showed a separation between OXLs serum levels of obese rats and ABX-treated rats in each photoperiod conditions. ABX-treated rats also showed a different profile compared to healthy rats. It is worth highlighting that the ABX effect was higher under L18 condition compared to both L6 and L12 conditions suggesting a stronger effect of long photoperiod (Figure 4a–c). Furthermore, when comparing photoperiod effects in ABX-treated rats, OXLs serum levels were more different in rats housed under L18 conditions compared to those housed under both L6 and L12 (Figure 4d).

### 3.3. GSPE Significantly Altered PGE2 Serum Levels in Obese Rats in a Photoperiod Dependent Manner

**Figure 5:**
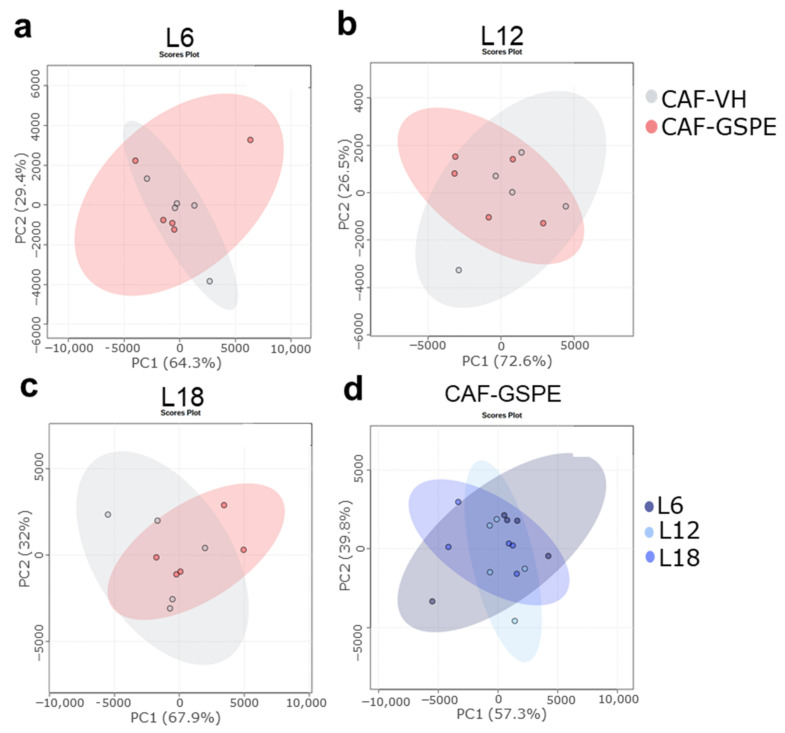
GSPE did not affect OXLs profile under any photoperiod condition. (**a**–**c**): GSPE effect in (**a**): L6; (**b**): L12; and (**c**): L18 conditions throughout principal component analysis (PCA); (**d**): Photoperiod effect on GSPE-treated rats. (*n* = 5).

GSPE decreased BW gain only under L18 condition. However, C-protein serum levels and the intake was not affected by GSPE administration (Figure S1). Regarding OXLs, GSPE only showed effect on PGE2 serum levels in obese rats under L6 conditions (Figure 2). PCA did not show any GSPE effect on the OXLs levels of CAF-fed rats in any photoperiod condition (Figure 5). Furthermore, when comparing GSPE-treated obese rats under different photoperiod conditions, any specific cluster was observed (Figure 5d).

### 3.4. GSPE Mitigated ABX-Mediated Changes in Serum OXLs Levels in CAF-Fed Rats in a Photoperiod Dependent Manner

Although GSPE did not affect C-reactive serum protein levels in obese rats treated with ABX (Figure S1), it significantly increased 15-Epi LXA4 and 6-keto PG1α levels under L18 conditions, mitigating ABX-mediated changes observed for these OXLs metabolites. Furthermore, an interaction effect of photoperiod, ABX and GSPE on these OXLs levels was observed (ANOVA: Ph × ABX × GSPE, *p* < 0.05). However, the combination of ABX + GSPE in obese rats did not show any effect on OXLs levels under either L6 or L12 conditions (Figure 2).

**Figure 6:**
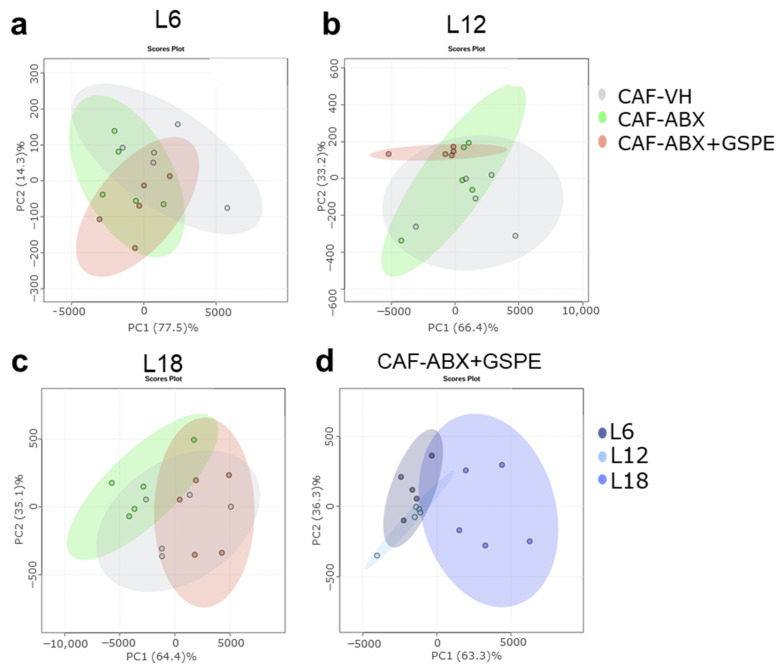
GSPE mitigated ABX-mediated effect on OXLs profile only under L18 conditions. (**a**–**c**): ABX + GSPE effect in obese rats in (**a**): L6; (**b**): L12; and (**c**): L18 conditions throughout principal component analysis (PCA); (**d**): Photoperiod effect on CAF-ABX + GSPE rats. (*n* = 5).

In addition, overall ABX + GSPE effects on OXLs levels in CAF-fed rat were analyzed using PCA. Thus, ABX + GSPE treated rats showed different effects depending on the photoperiod exposure. They showed a more different profile of OXLs compared to ABX-treated rats only under L12 and L18 conditions, whereas no changes were observed in rats under L6 (Figure 6a–c). Moreover, ABX + GSPE rats showed OXLs levels similar to obese rats only under L18 conditions, suggesting that GSPE treatment may mitigate the effects of ABX on OXLs profile under this photoperiod conditions (Figure 6a–c). Interestingly, overall OXLs profile of ABX + GSPE rats was different depending on photoperiod conditions, especially in rats exposed to L18 (Figure 6d).

### 3.5. LA and OA Were Affected by Diet and GSPE Differently Depending on Photoperiod Conditions

**Figure 7:**
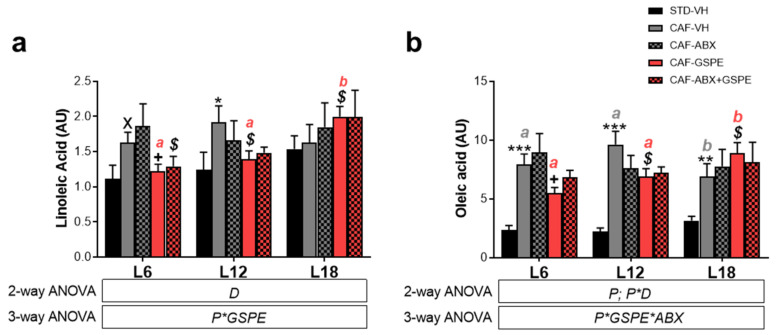
Diet, GSPE, and Photoperiod effect on LA and OA levels. (**a**) Linoleic acid levels; (**b**) oleic acids levels; * indicates significant diet effect between STD-VH and CAF-VH rats by 2-way ANOVA (factors: diet and photoperiod (P)) followed by LSD post hoc test (X *p* = 0.086; * *p* < 0.05; ** *p* < 0.01; *** *p* < 0.001); $ and *ab* indicate significant GSPE and Photoperiod effect, respectively, analyzed by 3-way ANOVA (factors: ABX, photoperiod, and GSPE) and followed by LSD post hoc test (*p* < 0.05). *ab* letters are shown in different color for each group. + indicates significant GSPE effect between CAF-VH and CAF-GSPE rats by *t*-test (*p* < 0.05). Data are plotted as the mean ± SEM (*n* = 7–8).

We also investigated the effects of GSPE, diet, and ABX in LA and OA, OXLs precursors. We found that CAF feeding affected LA and OA serum levels depending on the photoperiod conditions (P*D *p* < 0.05) (Figure 7). Thus, obese rats showed significant higher levels of LA under both L6 (*p* < 0.05) and L12 (*p* < 0.05) compared to healthy rats. However, obese rats under L18 did not show any diet effect, showing the same levels of these fatty acids than STD-fed rats (*p* > 0.05) (Figure 7a,b). In contrast, OA levels were significantly higher in obese rats compared to healthy rats independently of the photoperiod conditions (*p* < 0.05) (Figure 7b). In this context, OA levels showed an interesting photoperiod effect, their levels under L18 being lower in comparison with L12 conditions (*p* < 0.05) (Figure 7b).

GSPE effect was different depending on photoperiod conditions. Thus, GSPE significantly decreased OA and LA levels compared to obese rats under both L6 (*p* < 0.05) and L12 (*p* < 0.05) (Figure 7a,b), while the levels of these fatty acids were significantly increased under L18 conditions by GSPE compared to obese rats (*p* < 0.05) (Figure 7a,b). Accordingly, GSPE-treated rats showed an interesting photoperiod effect, LA and OL levels being significantly higher under L18 compared to both L6 (*p* < 0.05) and L12 (*p* < 0.05) (Figure 7a,b).

Additionally, when obese rats were treated with the combination of ABX and GSPE, LA levels were significantly decreased compared to ABX-treated rats only under L6 (Figure 7a) (*p* < 0.05). This GSPE effect in ABX-treated rats was not significant in OA, although a tendency to decrease was observed (*p* = 0.1) (Figure 7b). In contrast, ABX administration did not affect to LA and OA levels compared to obese rats, and it did not show any photoperiod effect either.

### 3.6. Correlations between OA and LA with OXLs Levels

**Figure 8:**
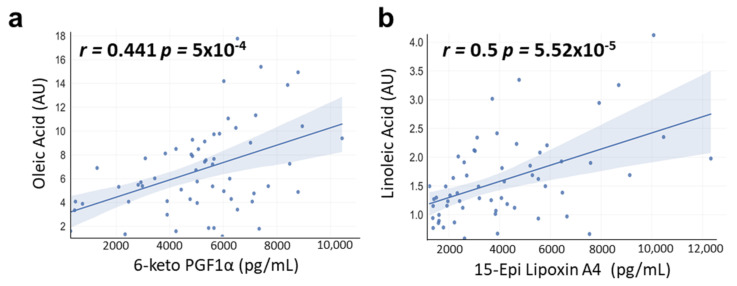
Correlations analyzed by Pearson’s rank correlation coefficient (r). Simple linear regression analysis of the significant observed correlation between: (**a**); 6-keto PGF1α and OA levels and (**b**) 15-Epi-Lipoxin A4 and LA levels d. (*n* = 7–8). AU: arbitrary units.

OXLs levels were positively correlated with OA and LA. Thus, 6-keto PG1Fα levels showed a significant positive correlation with the OA (*r* = 0.441 *p* = 5 × 10^−4^) (Figure 8a), while 15-Epi-LXA4 levels showed a significant positive correlation with the LA (*r* = 0.5 *p* = 5.52 × 10^−5^) (Figure 8b).

## 4. Discussion

OXLs constitute a large family of metabolites derived from fatty acid oxidation [17]. Among the multiple physiological processes in which they are involved, their role as key factors in the modulation of the immune response stands out [18,51]. For this reason, a growing number of studies point to them as potential biomarkers of chronic low-grade inflammation, playing a crucial role in diseases such as obesity [24,52]. Therefore, understanding the factors affecting OXLs metabolism is crucial to reduce the incident of these pathologies.

In this study, we demonstrate, for the first time to our knowledge, that photoperiod significantly influence OXLs metabolites in healthy and obese rats. These photoperiod-mediated changes in OXLs levels may be due to alterations in OXLs precursors’ metabolism. Hence, changes in photoperiod have been shown to partially disrupt clock genes signaling, impairing lipid metabolism [53,54]. Indeed, PUFAs from buccal mucosa samples have showed seasonal oscillations [55], suggesting that OXLs levels may also change depending on the season of the year. Moreover, photoperiod conditions have been reported to impact immune–endocrine interactions altering inflammatory processes, which may suggest that it may also alter inflammatory mediators such as OXLs [56].

Interestingly, photoperiods effects were more prominent in the case of obese rats. The more pronounced photoperiod effects in CAF-fed rats could be explained by a higher influence of these rhythms when altered metabolic conditions due to homeostasis disruption. Indeed, other parameters such as BW gain were not affected by photoperiod conditions in healthy rats while it significantly changed in obese rats [33]. Moreover, obese rats showed significant increased levels of C-reactive serum protein compared to healthy rats, indicating increased inflammation status. Additionally, significant differences were observed when comparing healthy and obese rats, which is in accordance with previous studies and could be explained by the different nutritional composition of the diets [25]. Thus, dietary intake of different PUFAs can modulate OXLs profile levels in the circulation and in different tissues [57,58]. Therefore, the differences in OXLs levels could be explained by the higher lipid content in CAF, including PUFAs, compared to STD. Interestingly, as mentioned before, these changes in OXLs levels between healthy and obese rats were different depending on the photoperiod conditions. Thus, for example 6-keto-PG1Fα, which has been related to anti-inflammatory effects, was increased in obese rats under L18, whereas no changes were observed in L6 or L12 conditions. 6-keto-PG1Fα acts as vasodilator, regulating vasomotor tone, reducing platelet aggregation, and inhibiting the recruitment and activity of inflammatory cells [59]. Moreover, it has been reported to act as a modulator of adipogenesis [60]. This photoperiod effect in this OXL is therefore in accordance with the increased BW and fat mass observed in these rats under L18 conditions [33] and with other studies where low levels of 6-keto-PG1Fα have been associated with reductions in both BW gain and epidydimal fat mass in high-fat diet-fed mice [61]. Finally, 15-Epi-LXA4 was decreased in obese rats under L18 conditions while no changes were observed in L6 or L12. 15-Epi-LXA4 is a lipid mediator derived from arachidonic acid, which has a strong anti-inflammatory and pro-resolution properties [62], being linked to attenuated obesity-induced adipose inflammation and decreased insulin resistance [63,64]. Hence, the decreased levels of this OXLs levels in obese rats under long photoperiod conditions is also in agreement with the increased obesity development observed in these animals [33].

Photoperiods effects in these OXLs levels could also be linked to changes in gut microbiota composition induced by photoperiods. Hence, it has been stablished that gut microbiota is involved in dietary PUFAs metabolism and that it significantly affect OXLs profile in both healthy and obese rats [25,29]. Moreover, gut microbiota composition can be influenced by photoperiod conditions [65,66,67]. In fact, our previous results suggested that obese rats housed under long photoperiod conditions have a significant different microbiota compared to those housed under either standard or short photoperiods, showing increased Proteobateria and Bacteroidetes levels, which have been linked with high inflammatory response [33,68]. Thus, the interaction between gut microbiota and photoperiod may also play a role in the differences in OXLs levels observed under the different photoperiod conditions. In this study, ABX treatment altered OXLs serum levels differently depending on the photoperiod conditions. Indeed, OXLs levels in ABX-treated rats were decreased and more similar to healthy rats than obese rats independently of photoperiod condition, suggesting that gut microbiota is a crucial factor in the metabolism of these lipid metabolites under obesogenic context, which is in accordance with our previous study [25]. In addition, ABX effect was higher under L18 condition compared to both L6 and L12 conditions, suggesting a stronger effect of long photoperiod. Therefore, these results suggested that both gut microbiota and photoperiod play an important role modulating the serum OXLs levels.

On the other hand, bioactive dietary compounds such as polyphenols have also been reported to influence OXLs metabolites. Thus, resveratrol affected OXLs biosynthesis in human neutrophils [30]. The dietary supplementation with polyphenol rich juice led to changes in urine OXLs profile in elite triathletes [32], and blueberry consumption mitigated OXLs generation during the recovery phase after physical exercise in humans [32]. Regarding proanthocyanidins, they are the most abundant polyphenols in human diet and they have shown to exert several health effects in obesity and associated pathologies, including anti-inflammatory properties [37]. In this study, GSPE effect was found only in PGE2 under L6 conditions, where it restored the levels of PGE2 observed in obese rats to the levels found in STD-fed rats. Interestingly, when rats were treated with the combination of ABX and GSPE, we found that GSPE mitigated ABX-mediated changes in serum OXLs levels in CAF-fed rats in a photoperiod dependent manner, showing a strong effect under L18 conditions. These results support our previous study, which reported that GSPE functionality in obesity is strongly influenced by L18 conditions through gut microbiota composition [36]. Therefore, these results suggest that polyphenols could regulate the OXLs metabolism influenced by the seasonal rhythms and gut microbiota, which may contribute to improve the low-grade of inflammation characteristic of obesity.

Finally, we investigated the effects of photoperiods in the levels of LA, precursor of OXLs metabolites, as well as in the levels of OA, which is a non-essential fatty acid which modulate PUFAs content in peripheral tissues such as liver and adipose tissue, influencing the levels of bioactive OXLs [40]. Overall, these fatty acids were increased in obese rats, probably due to their increased levels in this diet compared to healthy rats [69]. A photoperiod effect was only observed in the case of LA, which increased their levels in GSPE-treated obese rat under L18. OA levels were lower in obese rats, while GSPE increased their levels only in rats housed under L18 conditions. Hence, L18 conditions seem to be the most influencing photoperiod for these fatty acids. It is worth highlighting that, as mentioned above, this photoperiod was also where more pronounced changes in gut microbiota were observed together with increased BW and fat accumulation [33]. Moreover, 6-keto-PG1Fα and 15-Epi-LXA4 were positively correlated with OA and LA, respectively. The positive correlation of OA on 6-keto-PG1Fα levels is in accordance with a previous study where OA treatment increased 6-keto-PG1Fα levels in vascular smooths muscle cells in vitro [70]. Therefore, these results indicate that the modulation of these fatty acids, which are precursors of the OXLs, by proanthocyanidins and even by the diet could regulate the OXLs metabolism in a photoperiod-dependent manner.

## 5. Conclusions

Three main preliminary findings were found in the present study. Firstly, photoperiod conditions affected OXLs levels in both healthy and obesogenic conditions. Secondly, gut microbiota influenced the changes of OXLs levels modulated by photoperiods. Finally, GSPE did not affect most of the studied OXLs but mitigated the effects of gut microbiota dysbiosis on these metabolites in a photoperiod dependent manner. Although further investigation with a higher number of OXLs metabolites is needed to better understand these interactions and the role of these factors on OXLs metabolism, photoperiods seem to be a key factor that must be take into account when OXLs are studied under an obesogenic context.

## Data Availability

The data presented in this study are available on request from the corresponding author.

## Supplementary Materials

The following supporting information can be downloaded at: https://www.mdpi.com/article/10.3390/nu15030707/s1, Figure S1: Effects of photoperiods and treatments on body weight gain and C-reactive protein.
