# Supplementary material for: Photoperiod Conditions Modulate Serum Oxylipins Levels in Healthy and Obese Rats: Impact of Proanthocyanidins and Gut Microbiota
Source: Nutrients. 2023 Jan 30;15(3):707. doi: 10.3390/nu15030707 (PMC9920779; doi:10.3390/nu15030707)
Supplement: Supplementary file 1 [file nutrients-15-00707-s001.zip › nutrients-2026809-supplementary.pdf]

Supplementary material

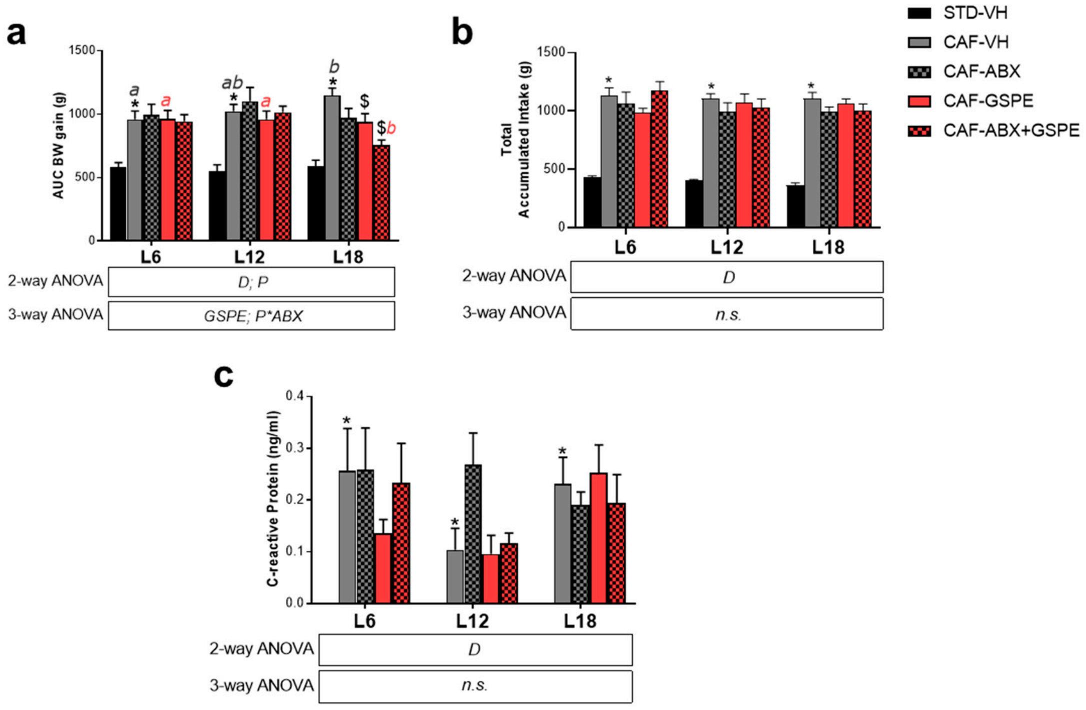

Figure S1. **Effects of photoperiods and treatments on body weight gain, intake and C-reactive protein**. (**a**): Area under the curve (AUC) of body weight (BW) gain. Figure adapted from Arreaza-Gil *et al.* 2022 [33,36]; (**b**): Total accumulated intake. Figure adapted from Arreaza-Gil *et al.* 2022 [33]; (**c**): c-reactive protein levels. Bar of the graph in STD-VH rats is not shown due to the levels were zero. * indicates significant diet effect between STD-VH and CAF-VH rats by 2-way ANOVA (factors: diet and photoperiod (P)) followed by LSD post hoc test; $ and *ab* indicate significant GSPE and Photoperiod effect respectively analysed by 3-way ANOVA (factors: ABX, photoperiod and GSPE) and followed by LSD post hoc test ($p < 0.05$). ab letter are shown in different color for each group. Data are plotted as the mean ± SEM (n = 7-8, except to total accumulated intake $n = 4$). L6: 6h light/18h darkness; L12: 12h light/12h darkness; L18: 18h light/6h darkness; STD: standard diet; CAF: cafeteria diet; VH: vehicle; GSPE: grape seed proanthocyanidin extract; ABX: antibiotic cocktail.
